# Gambling in Two Regional Australian Aboriginal Communities: A Social Practice Analysis

**DOI:** 10.1007/s10899-019-09858-9

**Published:** 2019-05-20

**Authors:** Sarah MacLean, Kathleen Maltzahn, Darlene Thomas, Andrew Atkinson, Mary Whiteside

**Affiliations:** 1 Centre for Alcohol Policy Research, La Trobe University, Melbourne, Australia; 2 School of Community and Clinical Allied Health, La Trobe University, Melbourne, Australia; 3 Mallee District Aboriginal Services, Mildura, Australia; 4 Gippsland and East Gippsland Aboriginal Cooperative, Morwell, Australia

**Keywords:** Indigenous, Gambling, Social practice, Interventions, Poker machines, Bingo

## Abstract

Reflecting international patterns, Aboriginal people in Victoria are more likely to gamble and to experience gambling harm than non-Indigenous Victorians. This paper describes experiences of gambling reported by 50 Aboriginal people interviewed in regional Victoria in 2016 and 2017 as part of studies initiated by two Aboriginal community-controlled organisations. Data were analysed using social practice theory (SPT) and coded to the elements of ‘meaning’, ‘material’, ‘competence’, and ‘temporality’. Across each element we identified highly contradictory experiences. Gambling held meaning as an opportunity for community gatherings but was also regarded as a cause of domestic violence, conflict, isolation and shame. Materially, the venues that offered gambling were experienced by many Aboriginal people as safe and welcoming, but at the same time gambling produced a damaging affective sense of addiction for some. Gambling was a competency that some people valued and taught to children, but it was also seen as undermining cultural practices. While Aboriginal people were historically denied access to licensed venues offering commercial gambling, many participants now found opportunities to gamble inescapable. The intermingling of benefits and harms described above supports the need for a multi-faceted response to gambling in Aboriginal communities, which includes harm reduction as well as supply restriction and treatment. Some experiences of gambling related by our participants reflected those reported also by non-Indigenous Australians, while others were differently nuanced. Because SPT is used to understand collectively-shared practices, it facilitates the identification of gambling interventions at the level of the community, as recommended by our research participants.

## Introduction

Aboriginal people in Australia are more likely to gamble than non-Indigenous Australians, reflecting an international pattern for Indigenous peoples (Hing et al. 2018; Bertossa and Harvey 2012). Eighty percent of Aboriginal participants in a survey conducted in 2011 had engaged in commercial gambling during the prior year, compared with 64% of Australians overall (Hing and Breen 2014). Little information is available on the extent of gambling harm in Australian Aboriginal communities, including economic costs (Hing et al. 2018; Bertossa and Harvey 2012). However, existing studies indicate that Aboriginal people experience disproportionate harm from gambling, with greater proportions of Aboriginal people meeting criteria for problem gambling than is evident in population groups (Hing et al. 2014b; Hare 2015). Research in the Australian state of New South Wales found that Aboriginal gamblers were over three times more likely (70% compared to 22%) to spend more than $100 per week on gambling than non-Aboriginal gamblers (cited in Bertossa and Harvey 2012). Aboriginal gamblers also appear more likely than other Australians to use electronic gaming machines; a modality strongly associated with harm, rather than participate in other forms of gambling (Hing and Breen 2014). Relatively few Aboriginal people seek treatment for gambling problems, preferring to rely on friends or family for help (Hing et al. 2014b).

Gambling is both popular and relatively normalised within some Australian Aboriginal and other Indigenous communities, and various reasons have been proposed to explain this (Williams et al. 2016; Holdsworth et al. 2013). Some forms of gambling have been recorded in Indigenous cultures prior to colonisation (Hing et al. 2014a; Gill et al. 2016; Belanger et al. 2017; Pasquaretta 1994). Gambling offers a promise of winnings, excitement, distraction from worries and problems, and important opportunities to socialise with family and other community members (Maltzahn et al. 2017, 2018; Breen et al. 2010; Cousins and Witcher 2004; Fiske 2015; Holdsworth et al. 2013).

As in mainstream communities, gambling by Aboriginal people has been attributed to adversity such as traumatic childhood events, unemployment, and social exclusion, as well as with mental health difficulties and alcohol and drug use. Gambling venues and companies drive gambling through promoting products and facilities and by locating venues in proximity to Indigenous gamblers (Dyall 2010). Problematic gambling in Aboriginal communities has also been linked to colonisation, oppressive government policies and the ongoing structural disadvantage that burdens Indigenous communities (Larsen et al. 2013; Hing et al. 2014a; McMillen and Donnelly 2008).

While they are by no means experienced by all Aboriginal people who gamble, negative impacts include poverty and debt, as well as stress, depression, feelings of guilt and shame, and strains on mental health (Breen et al. 2010; McGowan and Nixon 2004). Gambling losses can fuel interpersonal conflict, neglect of children, fragmentation of relationships and family violence (Dyall 2010; Nagel et al. 2011). In some cases gamblers resort to crime to find money to gamble or pay gambling debts (Breen et al. 2013). These harms flow onto Aboriginal families and communities (Hing and Breen 2014).

Despite this, little is documented of the place of gambling in Australian Aboriginal people’s social and cultural life. A recent review of literature identified ‘a dearth of research investigating gambling by Aboriginal people living in urban areas’ (Breen and Gainsbury 2012, p. 88) and the same could be said for those living in regional townships. The purpose of this paper is to develop an understanding of the social practice of gambling in two regional Aboriginal communities in the Australian state of Victoria. We draw on interviews about gambling conducted with 50 Aboriginal people. In interpreting these interviews, we use Social Practice Theory (SPT). SPT allows us to identify a range of elements of gambling practice and hence to show how suggested responses may be effective in minimising harms. By gambling, we refer to a range of activities including playing electronic poker machines (pokies), TAB betting (which facilitates racing and sports wagering), online sports betting, and cards and bingo where money is won and lost. We use the term ‘Aboriginal’ to refer to all Australian First Peoples, both Torres Strait Islander and Aboriginal. The term ‘Indigenous’ refers here to First Peoples across the world.

## Method

Two Aboriginal community-controlled health services, Mallee District Aboriginal Services (MDAS) and Gippsland and East Gippsland Aboriginal Co-operative (GEGAC), commissioned La Trobe University (LTU) to undertake collaborative exploratory research about gambling. MDAS is located in northern Victoria, in the Sunraysia region. GEGAC is in eastern Victoria. Although both studies concerned community experiences of gambling, they were different in focus. MDAS sought to understand the kinds of gambling played in their community, the nature of any associated harms, why relatively few community members accessed support services to deal with gambling, and what, if anything, community members believe needed to be done in response to gambling harms. GEGAC's project explored similar issues but focused specifically on experiences and impacts of gambling (both their own and that of others around them) for young Aboriginal people aged 16–25. Ethical approval to conduct the study was provided by LTU.

Our approach to designing and conducting the studies was collaborative and participatory (Pyett 2002). We sought to take a culturally safe and respectful approach that was consistent with guidelines for the conduct of research in Australian Aboriginal communities (National Health and Medical Research Council 2003). Initial meetings were held between staff of each agency and LTU to set broad parameters and protocols for the projects. After this we held longer workshops with all staff working on the project in each site, where we discussed research ethics, participant recruitment, interviewing, analysis and research feedback in each community. A semi-structured interview schedule for each study was trialled at these workshops, with many revisions made to ensure suitability to the communities concerned. The schedules included sections on experiences of gambling and gambling harms in the communities involved in the study, as well as asking participants for suggestions about effective interventions. Interviews were conducted in late-2016 to mid-2017. Some were conducted by LTU researchers in collaboration with agency staff, with later interviews conducted by agency staff alone.

Across the two sites we interviewed 50 Aboriginal people, recruited because they had personal experience of gambling, had been affected by another person’s gambling, or had professional experience working with gamblers. Twenty-six participants were accessed via MDAS and 24 via GEGAC. The study conducted with GEGAC included interviews with 8 young people aged 16–25. Participants were recruited by staff and through word-of-mouth. Workers were selected to give a range of perspectives and came from sectors including children’s welfare, community development, crime prevention, drug and alcohol, education, health, justice, mental health and youth work. Many workers interviewed had both personal (their own or family members’) and professional experience of gambling and reflected on both in interviews. Information about the interviewees and the number present at each interview is provided in Table 1.

**Table 1:** Information about interview participants

	MDAS	GEGAC	Total
Total interviewees	26	24	50
Adult community members	16	–	16
Young community members (aged 16–25)	–	8	8
Workers	10	16	26
Males	10	12	22
Individual interview	24	20	44
Pair interview	2	4	6

With participants’ permission, interviews were recorded and transcribed. Research analysis workshops were held with each agency, giving staff opportunities to elaborate on or clarify themes. A report was written with each agency which identified key issues associated with gambling and recommended a range of responses in each locality (Maltzahn et al. 2017, 2018). Later, a further workshop was held to discuss findings across the sites and consider the appropriateness of SPT to analyse the data, which forms the basis of this paper.

Quotes from participants used in this paper identify the location of the interviewee, their gender, and (for those for whom it is relevant) whether they were a professional working with Aboriginal people with gambling-related problems (worker) or aged 16–25 (young person). In analysing the interviews, we use SPT as outlined below.

## Social Practice Theory

Rather than understanding activities such as gambling as driven by individuals and their decisions, practice theorists regard social life as generated though the production and reproduction of patterned social behaviours. SPT as formulated by Shove et al. (2012) entails a ‘focus on the configuration of elements that establish [a practice] as a normal or necessary thing to do’ (Blue et al. 2016, p. 45). Thus SPT allows us to analyse how activities such as gambling occur within specific communities of people, and how harmful practices might be changed (Shove et al. 2012; Blue et al. 2016). SPT has been used productively to identify responses to practices such as substance use that can become habitual (Keane et al. 2017; Meier et al. 2017; MacLean et al. 2018).

In this paper we explore gambling practice in relation to four sets of elements commonly used in research informed by SPT. These are: ‘meaning’ (social and symbolic significance), ‘materials’ (objects, consumer goods and infrastructures), ‘competence’ (skills, practical know-how) and ‘temporalities (positioning in time). While Shove et al.’s (2012) formulation included only the first three of these, some researchers (Southerton 2006; Meier et al. 2017) have advocated for inclusion of ‘temporality’. This strikes us as particularly important in researching Indigenous health, as historical events such as colonisation, dispossession and harmful government policies have profound ongoing effects, and benefit payment cycles structure opportunities for recreation for people who are dependent on these sources of income.

To illustrate how this approach can be used to understand gambling practice: playing poker machines might require an expectation of possible financial gain or other benefit (meaning), a venue and machine (materials), knowing how to operate the machine (competence) and convenient venue opening hours (temporality). In the sections below, we show how this approach can be used to understand gambling in the settings of our studies. Each person who discussed gambling in an interview described different but overlapping sets of elements as part of their gambling practice. These sets of elements can be regarded as showing something of the social practice of gambling in two regional Victorian Aboriginal communities.

Our research team comprised Aboriginal and non-Aboriginal staff. We feel that SPT is an appropriate approach to Indigenous health research. It is consistent with the emphasis Indigenous researchers place on holistic approaches, where individual behaviour cannot be understood in isolation from the multiple social actors and forces involved, including families, communities, culture and wider structural inequalities, and where the role of researchers is to ‘bring knowledge systems and worldviews together’ (Laycock et al. 2011, p. 22).

## ‘Meaning’ Elements of Gambling Practice

For Shove et al. (2012) ‘meaning’ incorporates shared understandings of a practice and its effects. Gambling holds complex and sometimes contradictory meanings within the Aboriginal communities from which our interviewees were drawn. It was regarded as providing important opportunities for social engagement, bringing people together in an environment which usually felt safe and welcoming. Yet at the same time, gambling brought conflict, isolation from others and shame.

As we have written elsewhere in research on bingo (Maltzahn et al. 2019), research participants recognised gambling as a means to reinforce a sense of community. They emphasised that gambling provided opportunities for Aboriginal people to meet:

My nan goes all the time… she likes going because it’s sort of a socialising environment for other Aboriginal people too that they haven’t seen or someone other than family too they socialise with. [Sunraysia, female, worker]

The social aspect of gambling was particularly emphasised by older women, many of whom enjoyed playing commercial and community bingo. This provided them with time away from dealing with distressing problems in their family and respite from the care of grandchildren whom their children were not able to look after. For people who were socially isolated, gambling provided both entertainment and a valued social outing:

Cause she’s old, she’s in her sixties now, her husband left her and her kids are grown, and they’ve all gone their own ways and she’s more or less on her own. And she even says to me like ‘that’s me only comfort, that’s my only enjoyment in life I get now is going to the pokies’. Cause she hasn’t got her husband, she hasn’t got her kids around and she had pets but she lost her pets too. They’ve gone and died on her so she, she just wants that, that’s her comfort. [Sunraysia, male]

Some participants also regarded gambling, and in particular, bingo which is inherently more social than pokie playing, as beneficial in enabling people to stay away from alcohol and to spend time with family: ‘They’re not drinking and going out, they’re going to bingo with their mother and grandmother or whatever [Sunraysia, female, worker].

While bingo was often regarded as relatively benign, many identified the pokies as highly dangerous, relating personal experiences of harm. Although gambling provided opportunities to connect with family and community, participants spoke about how pokie-playing also eroded relationships. One young man regretted that his time with a family member was now taken up by sitting with him at the pokies where he was ‘like a zombie pressing that button and drinking a beer’ [Gippsland, male, young person]. Another worker [Sunraysia, female] spoke about how a relative would become angry with her when she refused to give her money to spend on the pokies. When money was lost, family conflict and violence could be among the consequences, as another woman described:

…if you’ve got the high [winning] side of it, they’re doing well, family prospers because we’ve got extra. But the minute it goes back the other direction then you’ve got anger issues, of course the family violence and also numbing agents, the drug and alcohol to try and suppress how you’re feeling ‘cause you’ve now just stuffed up and used the money. [Gippsland, female, worker]

People also spoke about how gambling generated self-disgust, as one young man explained: “I’m always just constantly fighting with myself … [Gambling]…makes you feel like an absolute shit, because that’s my family, that’s my [child], and I’m putting my addiction before [them]’ [Gippsland, male, young person]. Losing money could be very shameful, with one participant observing that this is the case for both Indigenous and non-Indigenous people:

You only hear about the wins. It’s only socially acceptable when you have a big win. When you’ve lost, it’s a shame job. You don’t want to tell anyone you’ve just blown $200. [Gippsland, female, worker]

It’s a shame thing. But I think that’s the broader community, I don’t think it’s just the Indigenous community. [Sunraysia, female, worker]

One of the impacts of shame about gambling losses that participants described was a reluctance to use services such as financial counselling: ‘Too embarrassed to go, shame or something, you know?’ [Sunraysia, female]. Participants in both locations emphasised the importance of developing holistic culturally-based community programs to counter these harms in the context of other issues in people’s lives, rather than individually-focused gambling-specific services. For example, and as we shall return to in concluding this paper, the introduction of yarning circles to share experiences of, and develop collective response to, problems such as gambling was recommended by participants in Sunraysia.

## ‘Material’ Elements of Gambling Practice

The material elements we consider here include the financial incentives to gamble, the poverty and deprivation that both leads to and results from gambling losses and the affective experience of addiction to playing poker machines. We describe how gambling venues are often places where Aboriginal people feel welcome, however the technology of poker machines is designed to produce overuse and a loss of control.

For many people, Aboriginal or not, the hope of financial gain is a powerful incentive to gamble. This is particularly so for those who live in poverty, as Aboriginal people disproportionately do. Research participants in both communities frequently spoke of gambling to alleviate financial stress and in the hope of securing some disposable income for ‘a quick fix or a quick buck’ [Sunraysia, male, worker]:

… I guess if you are unemployed you’re sort of going there with hope of making some money, it’s more of a necessity that you need to win that money. [Sunraysia, male, worker]

Hope to win big, hoping to get rich on one press. And I’ve seen people sit there and lost thousands…they bet high, yeah. So I’ve seen nieces in the hotel up the top there, and they spend all their pensions on it and the outcome of that is they’ve got no money when they go home, nothing. [Gippsland, female]

Some researchers have argued that Aboriginal people are less concerned at gambling-related financial loss than other Australians (Hunter (1993) in McMillen and Donnelly 2008). This did not seem to be the case in our research in regional urban contexts, perhaps because money lost from gambling flowed out of the community to corporations rather than staying within the community, as it is more likely to do when people play non-commercial card games. As evident above, the poverty that resulted from gambling losses caused conflict, deprivation and hardship for gamblers and for others around them.

In Indigenous cultures based on collectively and sharing of resources, gambling losses have a wide effect beyond the individual: ‘[Gamblers will] hit up other people, family members and uncles, aunties for money so the impacts you know, everyone bleeds off each other [Gippsland, male, worker]. This was also apparent in a reflection from the participant quoted below on her sadness after gambling away money that she wanted to share with family:

Cause I’m worried about, ‘I have to pay this, I got to pay that and how much am I going to have left and stuff it, only end up with 20 bucks left’ so it’s no use. You know sometimes if I got $20 in my bag and if my grandson walks in and he wants something I’ll give it to him, I’ll give him my last cause as long as I know he’s happy, I’m happy. But if I go and waste that money in that pokies, I’m very depressed cause I got no money, cause I done wrong. [Sunraysia, female]

Relatively few studies have explored the impact of gambling on children. A qualitative study conducted in Australia (not with Aboriginal people) found that children of problem gamblers regretted the absence of their parents, damage to their relationships, loss of trust and sense of security as well as material hardship (Darbyshire et al. 2001). Gambling can reduce parents’ availability to care for children and lead to neglect. Workers spoke of Aboriginal children going hungry when money was lost due to gambling:

…When they are losing they’ve got no food in the cupboards so they’re starving, don’t have food before they go to school. [The parents] know all that but they still want to go gamble. They’ve seen the bad side and they’ve seen the good side when they win cause they get things. But most of the time they’ve got nothing. [Gippsland, male, worker]

Aboriginal people living in regional Victoria have spoken about feeling unwelcome in public places including shops and licensed venues (MacLean et al. 2017). Many participants in our study said they were welcomed into gambling venues, at least while they had money to spend. As we shall discuss below under ‘temporality’, this must be understood in the context of exclusion of Aboriginal people from licensed venues in decades past. Participants spoke of comfortable couches, free tea and coffee, cheap food and being able to watch pay TV ‘…it’s paradise for people that doesn’t have much at home….’ [male worker, Gippsland]. Interviewees in Sunraysia told of a bus that picked people up to bring them to gambling venues, alongside other stories of personalised service:

… they roll the red carpet out for her, that sort of thing. That’s the thing with the club if you’re a regular, you know, they know you by your first name and then they treat you just like one of the family, and then, that’s how she feels, […] and now she’s a life member and then because she knows the staff that well and they treat her good. […] They make sure she is comfortable when she gets there. [Sunraysia, male]

Conversely, another participant commented that while the venues that host gambling welcome anyone regardless of their ethnicity, they asked him and other Aboriginal people to leave as soon as they had spent their money, saying: ‘”Out, you’re causing trouble, get out” […] I’m not sure about anyone else but it’s that way for the Aboriginal people’ [Sunraysia, male, worker].

Like alcohol and other drug use, gambling can be experienced affectively as a psychological and physical compulsion where people feel that their self-control is impaired. Poker machines are designed to block out other sensory input and induce people to stay for long periods of time (Livingstone et al. 2017). Playing pokies was widely regarded by research participants in both sites as physically and psychologically addictive, with associated loss of money and control producing a sense of shame as described in the section above:

I don’t know if it was the noises, the flashes, or whatever it was, but I think I was hooked from the moment I went in there. [Gambling on pokies] makes me feel good for the time I’m there. But at the same time I know I shouldn’t be, so I’m sort of torn between feeling good about being there but then feeling really shit because I know I’m wasting money. Mainly cause I’ve got [family responsibilities]. I’m always just constantly fighting with myself. […] It’s a terrible addiction. [Gippsland, male, young person]

Online forms of gambling are growing in their reach (Hare 2015). Others commented on pervasive opportunities to gamble online via mobile phones, with participants interviewed in Gippsland identifying this as the most common way that young people gambled. People found it particularly easy to spend money when it didn’t involve handing over cash:

I think it is, with those [online] sites, you don’t physically, you’re not physically spending your money, so you’re not keeping track of it. So that probably could be worse than going actually to the pokies cause there you’ve got a limit on how much you can spend in the venue or how much you can actually get out. Whereas if you are doing online gambling, you know you go until you’re broke. [Gippsland, male, worker]

## ‘Competence’ Elements of Gambling Practice

In SPT ‘competence’ refers to skills or practical know-how that enables people to engage in a social practice such as gambling. Participants spoke of gambling as a recognised strategy to deal with negative experiences and emotional states. They also described the skills involved in gambling, how these were taught to children, with some linking this to a loss of competence in cultural practices.

Gambling was widely understood as an effective way to alleviate boredom: ‘Well up here in the country they do it because there’s nothing else to do’ [Sunraysia, male]. It was also seen as helping people to manage negative emotions and cope with adverse experiences, albeit usually offering only temporary benefit:

It’s kind of like to do with a lot of depression, like, somebody feels depressed and they want to go get a drink or do some drugs, make them feel a bit better so pretty much gambling is the exact same. You’re feeling down and out so you just want to go and try your luck and see if you win some money and if you do you’re on cloud nine. And by the time it’s all gone then you’re back to feeling the same. [Gippsland, female, young person]

Participants in both communities spoke about how children in their communities learnt to gamble and to regard gambling as a routine aspect of life at a very young age. One suggested that gambling was taught to children so early it became part of who they were: ‘so it runs in their veins, in their blood’ [Sunraysia, male]. Others were critical of relatives who let children participate in card games:

I went up to New South for a while, and when I come back home, everyone was right into the cards you know […] A little girl walked in and I thought she was just, just having a sticky [looking] and when she sat up at the table and ‘Righteo’. And I said ‘Girl you’d better get out of here’ you know, she said ‘I’m allowed to play’ […] and then that caused a big fight between me and my cousin, ‘You leave my girl alone, she’s allowed to do’. ‘She’s not allowed, she’s an 11 year old.’ [Sunraysia, male]

It’s not the young fellas’ fault, it’s a learnt behaviour, they’ve learnt that at a young age, they saw it. By the time they were five or six they knew how to handle cards. [Gippsland, male, worker]

Some participants suggested that gambling took up space and time in people’s lives that they could otherwise use to develop competence in Aboriginal cultural practices. As two workers interviewed in Sunraysia described, culture had previously brought pleasure and meaning to Aboriginal people’s lives:

The older generation, of the generations because you know we’re having a battle with cultural stuff getting lost. We’re not practicing our cultural things as much as what we used to […] and that’s dying, whereas that’s where we used to get our enjoyment out of life […] I think that’s one of the impacts [of gambling], loss of culture. [Sunraysia, female, worker]

It’s sad to watch our culture sort of spiral down due to gambling, and to me, that’s what starts the stereotyping crap about: ‘That’s all black fellas do is gamble’. [Sunraysia, female, worker]

## ‘Temporal’ Elements of Gambling Practice

Gambling practice for Aboriginal people is structured temporally though historical forces, its availability across the day and patterning in relation to the social security benefit payment cycle.

From 1864 until 1957 Aboriginal people in Victoria were subject to legislation which denied them the right to enter licensed premises. Brady (2008) argues that this has had long term ramifications for Aboriginal people’s relationships with alcohol, promoting harmful patterns of use. It would be surprising if exclusion from licensed venues, where gambling often also occurs, did not have a similar effect on Aboriginal people’s gambling patterns. For example, we were struck by some older participants’ sense of appreciation for being welcomed into licensed venues as described above and wondered whether this was so keenly felt due to the contrast to their parents or grandparents’ exclusion from these same places. Moreover, when Aboriginal people were unable to access these venues, informal gambling became an important part of community life, as it continues to be today. Participants from each of the two regions recalled gambling involving card games and ‘two up’, a game where people bet on which side coins will land, as a key source of entertainment in Aboriginal communities during their youth:

I remember when I was younger at Lake Tyres [a mission where Aboriginal people were forcibly located] we’d play two up all night, under car lights. And then play poker all night then, outside, play two up and at dusk we’d turn the car lights on, so we could see the pennies drop, and inside playing cards all night. [Gippsland, male, worker]

Back in the old days before, oh well pokies were all big, it was the cards, cards. And the old girls, they would sit up for three or four days playing cards, you know, they wouldn’t leave that table until they won that jackpot in the middle. And then when they did win it, all out blue, ‘ah you ripped me off’. Whole community’s arguing over a card game. [Sunraysia, male]

In prominent contrast to their earlier exclusion from venues, participants reported that gambling opportunities, either at venues, or online, were now constantly available to them. A young man interviewed in Gippsland spoke of playing pokies for 10–12 hours, uninterrupted by venue workers or anyone else. For some, the only restriction on gambling was having money to spend, and these people would gamble as soon as they received their social security benefits. A worker from Sunraysia described clients turning up to a gambling venue around midnight after money went into their accounts and gambling until 3 am:

[…] soon as they get up, they’ve got their money. Even some people’s money [social security benefits] comes in at night time you know. They go get their money and go straight to [a gambling venue], pressing. [Sunraysia, male].

Participants in both communities argued strongly that restricting the availability of gambling venues and the hours of operation was a critical element in reducing the harms it caused. Those in Gippsland where the focus was on young people’s experiences argued strongly for regulation of online gambling and associated advertising.

## Discussion

Rather than viewing gambling as all bad or all good, Breen et al. (2010) suggests that gambling practices can be understood as a continuum, with unproblematic gambling at one end and problematic gambling at another. Our SPT analysis indicates that these positive and negative effects are intertwined and difficult to extricate from each other in the social practice of gambling in Aboriginal communities. In terms of the element of ‘meaning’, and consistent with other research, participants spoke of gambling as an opportunity to reinforce community in the tradition of card games. Yet gambling, and specifically playing pokies, was also regarded as causing conflict, domestic violence, and community fragmentation. Our analysis of the ‘material’ aspects of gambling suggests that venues offering gambling are sometimes experienced by Aboriginal people as welcoming and comfortable spaces. At the same time some identified these venues, as well as the wider settings where people gamble via the internet, as sites of racism and as producing addiction to gambling. Gambling was regarded as a ‘competency’ that Aboriginal people valued and hence some people spoke of children being taught to gamble at a young age. But the attention devoted to gambling was also thought to undermine cultural practices. Finally, in terms of ‘temporality’, while up until the late 1950s Aboriginal Victorians were denied access to licensed venues where commercial gambling occurred, our interviewees now felt that gambling was ever-present. For some, gambling was limited only by the availability of money to wager.

SPT offers a way to focus on the specific practices enacted within communities such as those from which our participants were drawn. Central to SPT is the notion that practices are dynamic. As Shove et al. write, practices: ‘emerge, persist and disappear as links between their defining elements are made and broken’ (2012, p. 21). Hence configurations of elements associated with harm can be altered, either deliberately though public health activity, or more informally as practices evolve over time. Below we suggest some ways that this might occur.

Changing meanings of gambling for Aboriginal people (or indeed for anyone) is a complex undertaking and our participants recommended providing opportunities for discussion within communities to reconsider the role of gambling in community life. While many gambling support programs and policies take an individual behaviour change approach (Miller et al. 2018), Breen et al. (2010) found that Aboriginal community members and gambling venue managers identified gambling as social, suggesting that existing interventions may over-emphasise individually-focused solutions. Participants in both communities included in this study argued for culturally-based community-focused responses. In Sunraysia, some recommended the introduction of yarning circles (Walker et al. 2013) where Aboriginal people could discuss gambling and develop collective responses. In themselves, yarning circles constitute an alternative social practice to gambling, yet fulfil many of the functions of gambling identified though the analysis above; offering meaningful connection to others, a sense of being welcomed into a safe place, opportunities to alleviate shame though discussing gambling and activity for people at times when little else is available.

Research increasingly underlines the structural determinants of harmful gambling. For example, the availability and density of gambling products, particularly of EGMs, is linked with gambling harm (Rintoul et al. 2013; Young et al. 2012; Strohäker and Becker 2018). In terms of our SPT analysis, this supports the need, as identified by our participants, to address material elements of harmful gambling by closing or restraining growth in gambling venues, limiting hours of access, and better regulating internet gambling and gambling advertising. It is also possible that if people had other ways to access money, gambling would become less appealing.

In terms of the element of ‘competence’, offering opportunities to engage in cultural activities and learn about culture was also identified by participants as a means of drawing people away from gambling. They felt that providing alternative activities would reduce people’s need to gamble to alleviate boredom or for distraction from life difficulties. Education about gambling harms and encouraging adults to teach games to children that don’t involve wagering may also shift the cultural position of gambling within Aboriginal communities over time.

The final element ‘temporality’ is evidently addressed in part by restricting gambling opportunities as mentioned above. A further need is to give people meaningful ways to spend time and engage with community. Bingo, especially when it is not played for money and therefore not technically gambling, seems to be less harmful than other games involving gambling yet still offers the sense of community connection that people yearn for (Maltzahn et al. 2019). Bingo where participants do not pay to play arguably constitutes a harm reduction approach to gambling.

Australian Aboriginal people negotiate two cultural worlds; engaging with mainstream social systems and places, while retaining distinct identities and cultures. It is unsurprising that many of the practices and effects of gambling reported by Aboriginal people in relation to gambling are also identified by non-Indigenous people. These include the appeal of winning money, opportunities to alleviate boredom and forget worries, but also a growing sense of dependence, loss of money and associated problems when gambling debts mount. Yet some gambling-related experiences appear in this and other studies to be differently nuanced for many Aboriginal people (Hing and Breen 2014). As we have noted, Aboriginal and other Indigenous peoples are more likely to gamble and to experience harms from gambling than non-Indigenous people (Hare 2015; Delfabbro 2012). Yet Aboriginal people’s experiences of gambling also differ in ways that cannot be captured by statistics. Aboriginal people speak of the significance of being welcomed into gambling venues in a context where their parents or grandparents were excluded from licensed settings in the past. Because of this card-playing was, and continues to be, an important pastime within communities, which is reflected in gambling’s ongoing meaning as a means to connect with community. Gambling harms are overlaid by historical events, perpetuating disadvantage though entrenching poverty and (in some cases) contributing to intergenerational trauma through leading to poorer parenting practices and erosion of culture. In a culture where resources are shared, shame about getting into trouble and letting down family or not having money to give to others seems particularly acute for Aboriginal people.

This is a qualitative study of gambling in two regional Victorian Aboriginal communities and an evident limitation is that it is unclear how widely our findings may be generalised. It seems likely that Aboriginal people in other regional Australian communities and many urban centres share many of the experiences described here (Hing and Breen 2014; Williams et al. 2016). While we do not wish to generalise our findings to Indigenous people in other countries, the shared history of exclusion from mainstream venues and institutions, alongside ongoing discrimination and trauma from colonisation indicate that there commonalities in Indigenous experiences of gambling, and this is supported in a range of studies (Gill et al. 2016; Belanger et al. 2017; Pasquaretta 1994; Larsen et al. 2013; McMillen and Donnelly 2008; Williams et al. 2016; Dyall 2010).

Public health frameworks which focus on addressing structural elements of gambling harm may not on their own be sufficient to meet the needs of Indigenous and disadvantaged communities, as the specificities of their experiences can be lost in a focus at the level of population (Breen et al. 2013). The intermingling of benefits and harms described above supports the need for a multi-faceted response to gambling in Aboriginal communities, which includes harm reduction to address the likelihood that people will continue to gamble into the future. Aboriginal people in our study argued for structural or supply level responses to gambling, including efforts to restrict availability of pokies and regulate emerging internet gambling sites. While existing counselling and financial management services were regarded as a necessary part of the response, participants also recommended the introduction of community-level responses to gambling that are grounded in Aboriginal culture and that address historical and socioeconomic experiences of Victorian Aboriginal communities.

We found SPT to be a helpful tool in mapping gambling practices in communities and displaying these elements visually to support our discussions. Rather than imagining gambling as driven predominantly by individuals and their choices, SPT pushes researchers to consider cultural forces, skills and also how things and places (i.e. poker machines and betting venues) produce practices such as gambling. Because SPT is used to describe collectively-shared practices, it facilitates identification of gambling interventions that are targeted at communities. Thus, it provides a method for conducting research with Aboriginal people that recognises the complex aetiologies of social concerns, and identifying suggestions for collective action to address them.
